# Submucosal Tunnelling Endoscopic Resection for Oesophageal Duplication Cyst: A Case Report

**DOI:** 10.34172/mejdd.2025.436

**Published:** 2025-07-30

**Authors:** Srinu Deshidi, Gaurav Mahajan, Viswanath Kamisetty, Spoorthi Kolla, Venu Gongati, Gongala Harshavardhan Reddy, Bhaskar Kante, Sreekanth Appasani

**Affiliations:** ^1^Krishna Institute of Medical Sciences, Hyderabad, India; ^2^Indian Naval Hospital Ship (INHS) Asvini, Mumbai, India

**Keywords:** Oesophagus, Duplication cyst, STER, Dysphagia

## Abstract

Oesophageal duplication cysts are rare congenital anomalies of the foregut. Although frequently asymptomatic, these cysts may cause symptoms like dysphagia, chest pain, or respiratory issues if they grow large enough to compress adjacent structures. Endoscopic ultrasound is essential for accurate diagnosis, and though surgical resection remains the conventional therapy for symptomatic cysts, advanced endoscopic techniques are increasingly recognized as effective, less invasive alternatives. We report the case of a 40-year-old man who presented with dysphagia to solids for 3 months. Upper gastrointestinal endoscopy revealed a large submucosal lesion with fluctuation sign positive, and EUS demonstrated a 4×6.5 cm anechoic to hypoechoic lesion arising above the muscularis propria, likely an oesophageal duplication cyst. The patient underwent successful submucosal tunnelling endoscopic resection (STER) of a cystic lesion under general anaesthesia. This case highlights the feasibility and efficacy of STER as a minimally invasive treatment option for oesophageal duplication cysts.

## Introduction

 Oesophageal duplication cysts are rare congenital anomalies of the foregut that constitute 0.5% to 2.5% of all oesophageal masses.^1^ Most of the duplication cysts are asymptomatic, but some patients present with dyspnoea, chest pain, or dysphagia.^2^ A diagnosis may be made with a combination of imaging modalities (endoscopy, barium swallow, chest computed tomography (CT)/magnetic resonance imaging (MRI), and endoscopic ultrasound (EUS). EUS has been widely used as a modality for the evaluation and diagnosis of duplication cysts.^3^ The standard management for symptomatic cysts is surgical resection. Endoscopic minimally invasive treatment management is a reasonable alternative to a surgical approach.^4^ We present a case of oesophageal duplication cyst successfully managed by the endoscopic approach.

## Case Report

**Figure 1:**
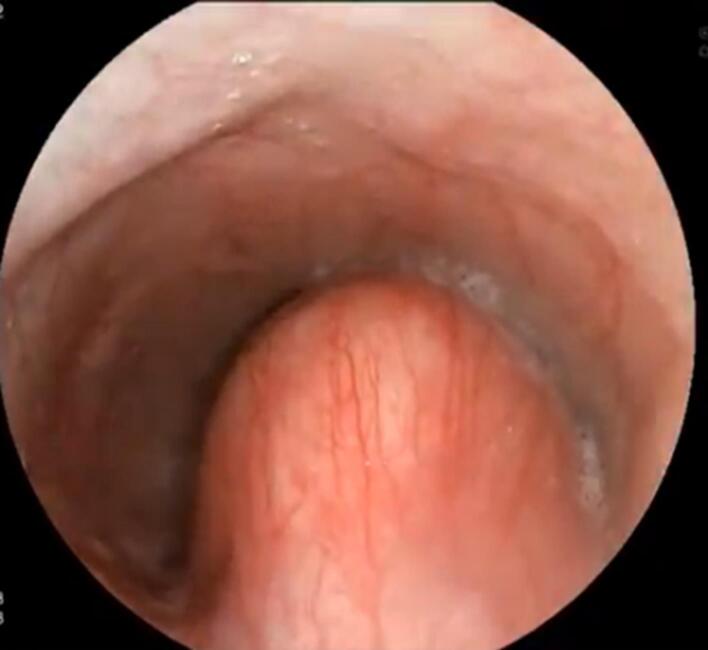
Endoscopy showing a large submucosal lesion in the mid-oesophagus

**Figure 2:**
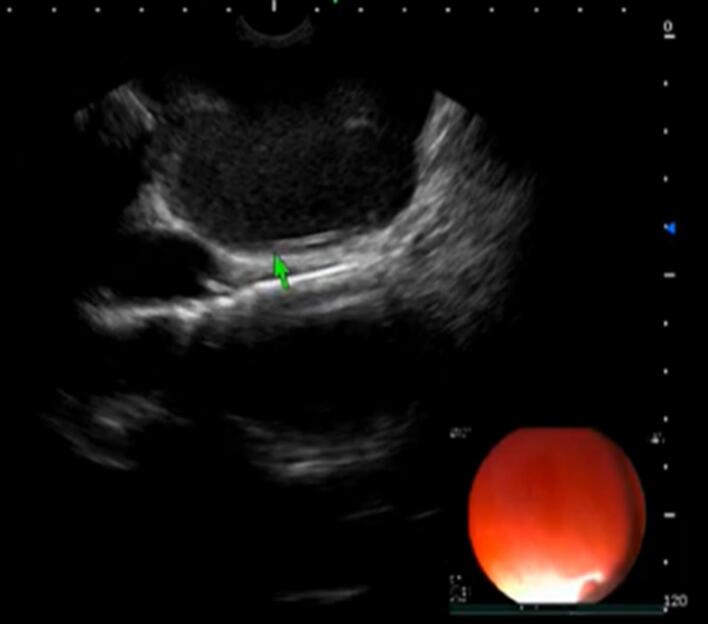
Endoscopic ultrasound showing hypoechoic to anechoic lesion in the mid-oesophagus

 A 40-year-old man presented with dysphagia to solids for the last 3 months. He had no history of weight loss, decreased appetite, odynophagia, reflex symptoms, or pill intake. Upper gastrointestinal (UGI) endoscopy was done, which was suggestive of a large submucosal lesion (Figure 1). The lesion demonstrated a positive fluctuation sign when probed with biopsy forceps. Subsequent EUS examination was done, suggestive of an anechoic to hypoechoic lesion of size 4 × 6.5 cm, originating above the muscularis propria was noted (Figure 2).

**Figure 3:**
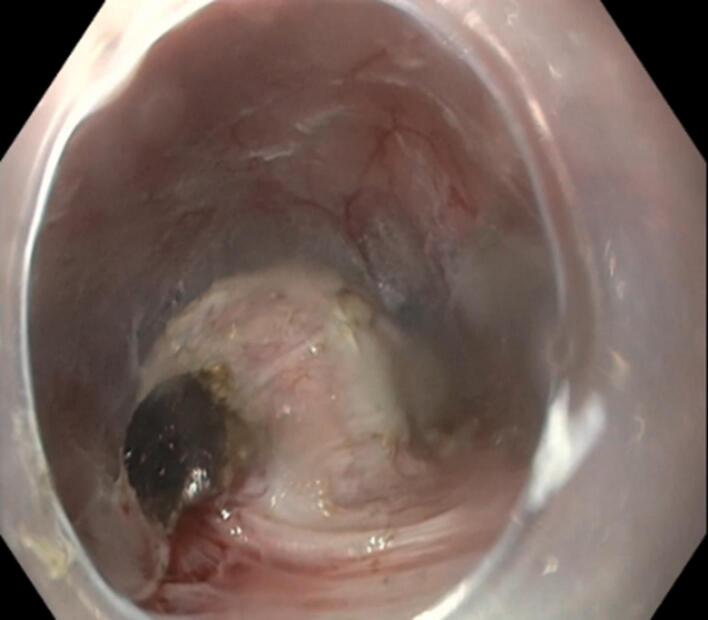
Submucosal tunnel showing a lesion

**Figure 4:**
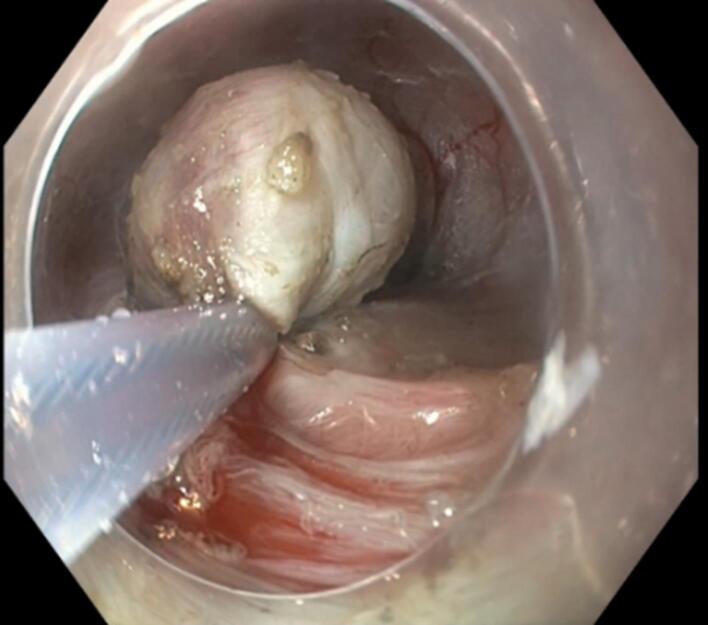
Lesion captured with polypectomy snare

**Figure 5:**
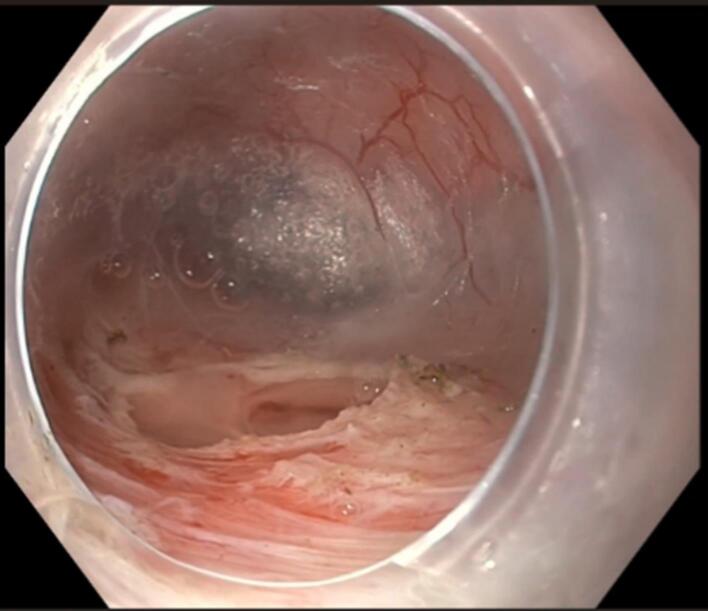
Post resection

 Under general anaesthesia, submucosal tunnelling endoscopic resection (STER) was performed. In this procedure, a small mucosal incision (~1.5–2 cm) was made 5 cm proximal to the lesion. A methylene blue-mixed saline solution was injected into the submucosal space to create a cushion and facilitate dissection. Using an electrosurgical knife, a straight submucosal tunnel was made until the lesion was visible (Figure 3). The lesion was then dissected from the surrounding tissue with the electrosurgical knife, followed by resection using a hot snare (Figures 4 and 5). During resection, thick, viscous, yellowish fluid drained from the cyst. Finally, the tunnel was closed at the incision site using clips. Histopathological examination was suggestive of oesophageal duplication cyst. The patient was kept nil per oral overnight post-procedure, followed by a liquid diet for one week, and then gradually advanced to a regular diet.

## Discussion

 Oesophageal duplication cysts are rare congenital foregut anomalies that arise due to incomplete vacuolization of the embryonic foregut during the 3rd to 4th week of gestation.^5^ The prevalence of oesophageal duplication cysts is 0.01%, representing 10-15% of all GI duplication cysts, with a male predominance.^6^ Approximately two-thirds of oesophageal duplication cysts are located in the lower third of the oesophagus, while the remaining one-third are found in the upper or middle third.^7^ Oesophageal duplication cysts are usually solitary, though they can occasionally be multiple. Only about 10% of these cysts have a connection with the adjacent oesophageal lumen.^8^ Histologically, the cyst lumen is lined with stratified squamous epithelium or embryonic pseudostratified epithelium and is distinguished by the presence of two smooth muscle layers ^9^ About 80% of duplication cysts are diagnosed in childhood, and the remaining are diagnosed in adulthood.^10^ Our case had a solitary cyst and was diagnosed in adulthood. Patients with oesophageal duplication cysts are often asymptomatic but may experience symptoms like dysphagia, cough, stridor, or chest pain due to compression of nearby structures. Proximally located cysts can lead to respiratory symptoms, while distally located cysts are more likely to cause dysphagia. It can also lead to complications such as infection, bleeding, rupture, and malignant transformation within the cyst.^11,12^ Oesophageal duplication cysts are sometimes associated with other congenital abnormalities, such as intestinal duplications or atresia, vertebral anomalies, and others. It is important to exclude other associated anomalies in patients with congenital abnormalities. Our patients had no associated anomalies. Endoscopy helps determine the cyst’s location in relation to the upper and lower oesophageal sphincters, as well as in examining the overlying mucosa, which is typically normal. A barium swallow is effective in pinpointing the exact location of the lesion and its relationship to the oesophageal hiatus. EUS can assess both the intramural and extramural relationships with the oesophagus and differentiate between solid and cystic lesions.^13^ In EUS it appears as homogenous hypoechoic mass with multi-layered wall and well-defined margins or as anechoic cyst. It is important to note that many duplication cysts may present with an atypical appearance and can resemble other pathological conditions, including malignant adenopathy.^3^ In our case in EUS it appears as anechoic to hypoechoic lesion originating above the muscle layer.

 Treatment options for symptomatic oesophageal duplication cyst include surgical resection and minimally invasive endoscopic management. For an asymptomatic cyst, treatment options include surgical resection or endoscopic therapy and sometimes simple observation. Endoscopic fenestration and STER are minimally invasive endoscopic treatment options.^14,15^ The index patient underwent successful STER under general anaesthesia. As a sophisticated third-space endoscopic procedure, STER requires considerable technical expertise and is typically reserved for subepithelial lesions. While effective, its use in managing oesophageal duplication cysts remains relatively novel, with only sporadic case reports published to date.^14,16^

## Conclusion

 Oesophageal duplication cysts, though rare, should be considered in the differential diagnosis of submucosal oesophageal lesions, especially in patients presenting with dysphagia or chest symptoms. While surgical resection has traditionally been the mainstay of treatment for symptomatic cysts, minimally invasive endoscopic techniques like STER offer a safe and effective alternative with excellent outcomes and reduced morbidity.
